# Fiber Type-Specific Nitric Oxide Protects Oxidative Myofibers against Cachectic Stimuli

**DOI:** 10.1371/journal.pone.0002086

**Published:** 2008-05-07

**Authors:** Zengli Yu, Ping Li, Mei Zhang, Mark Hannink, Jonathan S. Stamler, Zhen Yan

**Affiliations:** 1 Department of Medicine, Duke University Medical Center, Durham, North Carolina, United States of America; 2 Department of Biochemistry, Duke University Medical Center, Durham, North Carolina, United States of America; 3 Duke-NUS Graduate Medical School, Singapore, Singapore; 4 Department of Biochemistry, Life Science Center, University of Missouri-Columbia, Columbia, Missouri, United States of America; Baylor College of Medicine, United States of America

## Abstract

Oxidative skeletal muscles are more resistant than glycolytic muscles to cachexia caused by chronic heart failure and other chronic diseases. The molecular mechanism for the protection associated with oxidative phenotype remains elusive. We hypothesized that differences in reactive oxygen species (ROS) and nitric oxide (NO) determine the fiber type susceptibility. Here, we show that intraperitoneal injection of endotoxin (lipopolysaccharide, LPS) in mice resulted in higher level of ROS and greater expression of muscle-specific E3 ubiqitin ligases, muscle atrophy F-box (*MAFbx)/atrogin-1* and muscle RING finger-1 (*MuRF1*), in glycolytic white vastus lateralis muscle than in oxidative soleus muscle. By contrast, NO production, inducible NO synthase (*iNos*) and antioxidant gene expression were greatly enhanced in oxidative, but not in glycolytic muscles, suggesting that NO mediates protection against muscle wasting. NO donors enhanced *iNos* and antioxidant gene expression and blocked cytokine/endotoxin-induced *MAFbx/atrogin-1* expression in cultured myoblasts and in skeletal muscle *in vivo*. Our studies reveal a novel protective mechanism in oxidative myofibers mediated by enhanced *iNos* and antioxidant gene expression and suggest a significant value of enhanced NO signaling as a new therapeutic strategy for cachexia.

## Introduction

Chronic diseases are often associated with and exacerbated by loss of lean body mass known as cachexia mainly due to skeletal muscle wasting. Recent research efforts have led to the current understanding of the molecular and signaling mechanisms responsible for skeletal muscle atrophy under various pathological conditions [1]–[7]. In particular, enhanced expression of muscle-specific E3 ligases, *MAFbx/atrogin-1* and *MuRF1*, with function in protein ubiquitination have been shown to mediate proteosome-dependent protein degradation in muscle wasting. Therefore, expression of these genes is now served as an early marker of skeletal muscle atrophy. Skeletal muscles of different fiber type composition have different contractile and metabolic properties. Oxidative muscles (predominantly type I and/or IIa fibers) are generally rich, while glycolytic muscles (predominantly type IId/x and/or IIb fibers) are poor, in mitochondria and capillaries. Interestingly, oxidative muscles are more resistant to atrophy than glycolytic muscles [8]–[13]; however, much remains unknown regarding the molecular mechanism(s) for the fiber type-specific muscle wasting. The “built-in” protection in oxidative myofibers provides an excellent experimental model to decipher the skeletal muscle wasting process and search for new drug target(s) to treat this detrimental symptom that affects the morbidity and mortality of many chronic diseases.

ROS are well recognized to have primarily deleterious effects in mammalian cells. High level, sustained increase in ROS leads to oxidative cellular injury that plays “cause-effect” roles in the pathogenesis of chronic diseases and syndromes including cachexia. The deleterious role of ROS in muscle wasting has been shown in previous studies. Firstly, increased production of ROS [14]–[17] and reduced antioxidant gene expression [18] are associated with muscle catabolism. Secondly, exogenous ROS activate proteosome-dependent protein degradation [6]. Lastly, antioxidant treatment attenuates skeletal muscle atrophy [19], [20]. However, little is known about the fiber type differences of oxidative stress in skeletal muscle in cachexia.

The functional role of nitric oxide (NO) and related oxides of nitrogen (reactive nitrogen species, RNS) in skeletal muscle wasting is controversial and far less well understood except that it is well known that NO synthase (NOS) expression is often increased in muscle wasting disorders [11], [21]–[25]. On the one hand, NO and RNS have been shown to acutely inhibit mitochondrial respiration and muscle contractile function [21], [25]–[27]; thus a scavenger of ROS-RNS [23] or NOS inhibitor [25] prevents muscle dysfunction. On the other hand, iNOS expression has been shown to increase dramatically in disease-resistant oxidative muscles following pathological challenges, and iNOS inhibition increases disease susceptibility [11]. Furthermore, chronic inhibition of NOS resulted in muscle dysfunction and atrophy [28]. In light of these apparently conflicting findings, the functional role of NO and its derivatives in skeletal muscle wasting remain to be determined. In particular, there has not been an attempt to define the functional relationship between ROS and NO in skeletal muscle under the catabolic condition.

We have recently obtained comprehensive physiological, morphological, biochemical and gene expression evidence in a mouse model of chronic heart failure to indicate that oxidative myofibers are more resistant than glycolytic myofibers to chronic heart failure [12]. Consistently, there are significant differences between these myofibers in their atrophic response to cachetic stimuli, such as LPS and TNF-α. To improve our understanding of the mechanism(s) underlying the protection associated with oxidative phenotype, we designed this study to focus on fiber type-specific differences in ROS and NO and to determine if NO plays a functional role of muscle atrophic process related to cachexia. We obtained evidence that oxidative muscles had greater production of NO and expression of antioxidant genes, and endure lower oxidative burden than glycolytic muscles in response to cachectic stimuli. We then tested the hypothesis that up-regulation of antioxidant genes were dependent on NO, and thereby provided the protection from cachectic stimuli. This hypothesis was confirmed both in cultured muscle cells and in intact skeletal muscle *in vivo*. Results from this study provide potential mechanistic insights into the relatively greater resistance to cachexia associated with oxidative phenotype in skeletal muscle.

## Results

### Endotoxin induces less ROS production and muscle-specific E3 ligase expression in mouse oxidative soleus muscle than in glycolytic white vastus lateralis muscle

**Figure 1:**
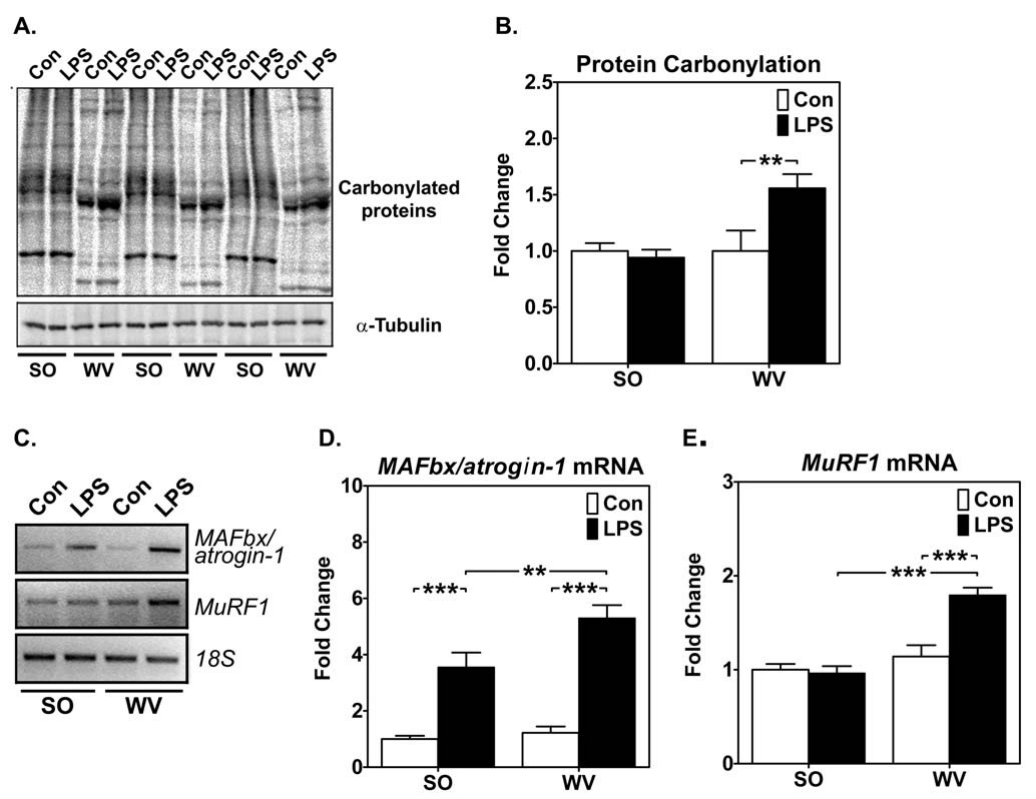
Endotoxin induces less oxidative stress and proteosome-dependent protein degradation in mouse oxidative than in glycolytic muscles. Whole muscle homogenates and total RNA from oxidative soleus muscles (SO) and glycolytic white vastus lateralis muscles (WV) 12 hours after intraperitoneal (i.p.) injection of LPS (1 mg/kg body weight) were assayed for the carbonyl groups by immunoblot analysis (A and B) and for *MAFbox/atrogin-1* and *MuRF1* mRNA expression by semi-quantitative RT-PCR (C, D and E). A) Immunoblots show total protein carbonylation (top panel) after LPS or saline injection (Con) with α-tubulin (bottom panel) as loading reference. Each lane was loaded with 20 µg of proteins, and data were shown in triplicates; B) Quantification and comparison of protein carbonylation (n = 12; ** *P*<0.01); C) Gel images show semi-quantitative RT-PCR analysis for *MAFbx/atrogin-1* and *MuRF1* mRNA expression with *18S* ribosomal RNA as control; and D) and E) Quantification and comparison of *MAFbx/atrogin-1* and *MuRF1* mRNA expression. The SO muscles from saline injected mice were set as reference (n = 6; ** and *** *P*<0.01 and 0.001, respectively).

Oxidative myofibers are resistant to cachectic stimuli induced by chronic heart failure and inflammatory cytokines [12]. One possible explanation is that systemic inflammation induces less oxidative stress in oxidative than in glycolytic myofibers. To test this hypothesis, we injected mice with endotoxin and measured protein carbonylation as an index of oxidative stress. LPS injection (12 hours) did not result significant changes in carbonylated proteins in soleus muscles, but resulted in a significant (∼50%) increase in white vastus lateralis muscles (Fig. 1A and 1B). LPS induced greater expression of muscle-specific E3 ligase *MAFbx/atrogin-1* [1], [3], [4] in white vastus lateralis muscles than in soleus muscles (Fig. 1C and 1D). LPS also significantly increased *MuRF1* expression in vastus lateralis muscles, but not in soleus muscles (Fig. 1C and 1E). These findings are completely consistent with our previous findings [12]. Thus, endotoxin challenge results in significantly less oxidative stress, hence less activation of the proteosome-dependant protein degradation, in oxidative than in glycolytic muscles. We have also observed similar changes in a mouse model of sepsis induced by S. aureas infection (unpublished results). Therefore, the fiber type-specific muscle atrophic response appears to be relevant to various cachectic conditions that can be recapitulated by endotoxin injection.

### Endotoxin induces greater NO production and iNOS expression in mouse oxidative soleus muscle than in glycolytic white vastus lateralis muscle

**Figure 2:**
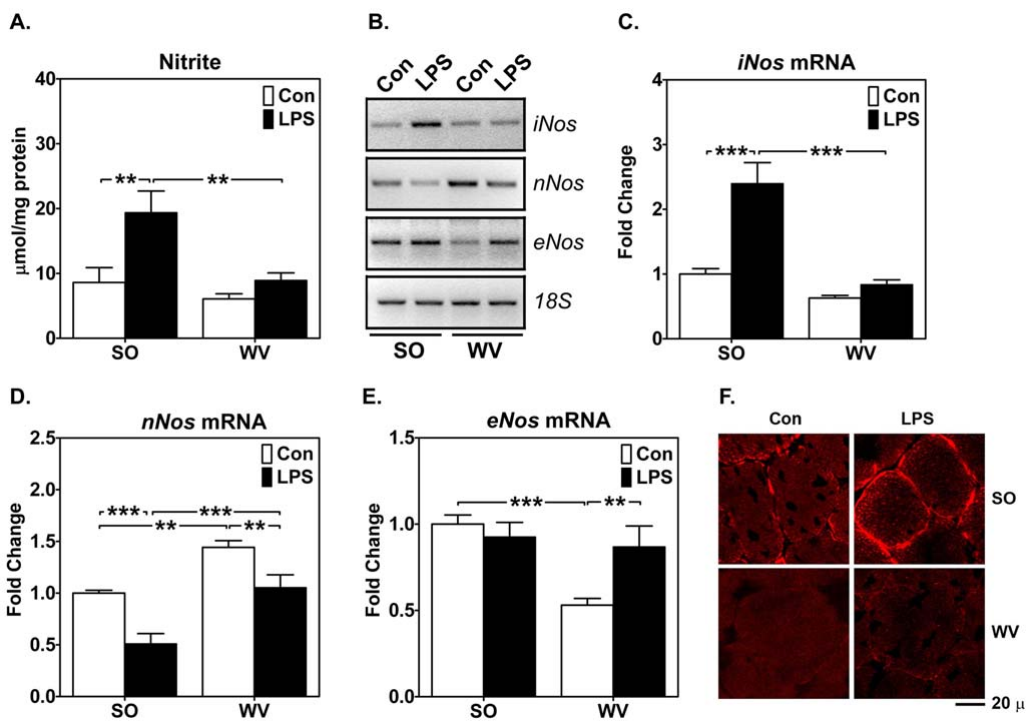
Endotoxin induces greater iNOS expression and NO production in mouse oxidative than in glycolytic muscles. Whole muscle homogenates, total RNA and muscle sections from SO and WV after LPS or saline injection were assayed for nitrite (A), *iNos*, *nNos* and *eNos* mRNA expression by semi-quantitative RT-PCR (B,C, D and E), and iNOS protein expression by immunofluorescence (F). A) Quantification and comparison of nitrite concentration (n = 5–6; ** *P*<0.01); B) Gel images show levels of *iNos*, *nNos* and *eNos* mRNA expression with *18S* ribosomal RNA as control; C), D) and E) Quantification and comparison of *iNos*, *nNos* and *eNos* mRNA expression. The SO muscles from saline injected mice were set as reference (n = 9; ** and *** *P*<0.01 and 0.001, respectively); and F) Immunofluorescence staining for iNOS protein after LPS or saline (Con) injections (12 hours), showing higher level of basal and induced expression of iNOS in SO vs. WV muscles.

To understand the possible functional role of NO-dependent signaling/gene regulation in fiber type specificity of muscle wasting, we assessed NO production in both soleus and white vastus lateralis muscles by measuring the stable NO product, nitrite. Nitrite concentration was not significantly different between these two muscles under basal conditions. However, LPS injection led to ∼100% increase in nitrite concentration in soleus muscles, but not in white vastus lateralis muscles (Fig. 2A). The increase in nitrite in soleus muscle was associated with an enhanced iNOS expression (Fig. 2B, 2C and 2F) with majority of protein confined to the periphery of oxidative myofibers. In contrast, neuronal NO synthase *(nNos)* expression was reduced in both soleus and white vastus lateralis muscles (Fig. 2D), whereas endothelial NO synthase *(eNos)* expression was low in white vastus lateralis muscles under basal conditions and was induced to a level comparable to that in the soleus muscles (Fig. 2E) following LPS injection. These findings suggest that oxidative myofibers have great inducibility in NO production, probably through induced expression of iNOS.

### Endotoxin induces greater antioxidant gene expression in oxidative soleus muscle than in glycolytic white vastus lateralis muscle

**Figure 3:**
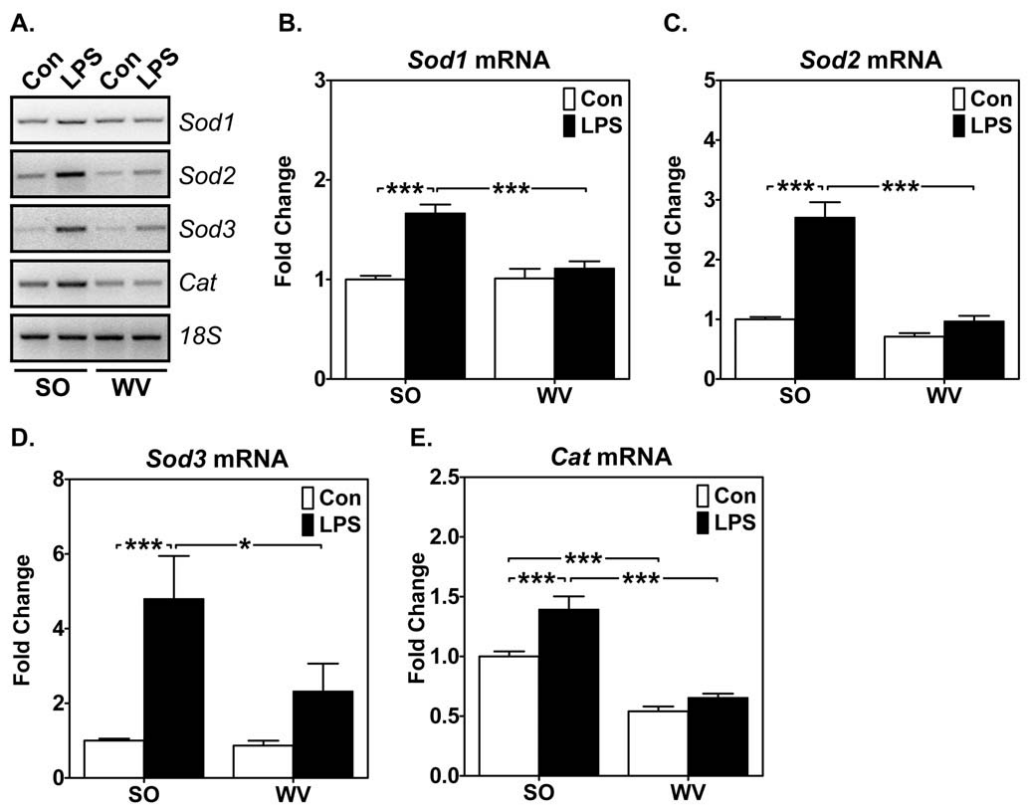
Endotoxin induces greater antioxidant gene expression in mouse oxidative than in glycolytic muscles. Total RNA from SO and WV after LPS or saline (Con) injection were assayed for antioxidant gene expression by semi-quantitative RT-PCR. A) Gel images show levels of *Sod1*, *Sod2*, *Sod3 and Cat* mRNA expression with *18S* ribosomal RNA as control; and B), C), D) and E) Quantification and comparison of *Sod1*, *Sod2*, *Sod3 and Cat* mRNA, respectively (n = 9; *, ** and *** *P*<0.05, 0.01 and 0.001, respectively).

Our finding of less oxidative stress in soleus muscles than white vastus lateralis muscles following endotoxin challenge prompted us to determine if enhanced antioxidant gene expression contributes to the protection of oxidative myofibers. We performed semi-quantitative RT-PCR analysis for mRNA of the antioxidant genes: superoxide dismutases (*Sod1, Sod2* and *Sod3*) and catalase (*Cat*). LPS injection resulted in significantly increased expression for all four antioxidant genes in soleus muscles with no or less increases in white vastus lateralis muscles (Fig. 3A–E). We have, therefore, obtained evidence for differentially enhanced antioxidant gene expression in oxidative vs. glycolytic myofibers that are likely intrinsic to the muscles.

### NO donor blocks TNF-α-induced muscle-specific E3 ligase expression and enhances iNOS and antioxidant gene expression in cultured muscle cells

**Figure 4:**
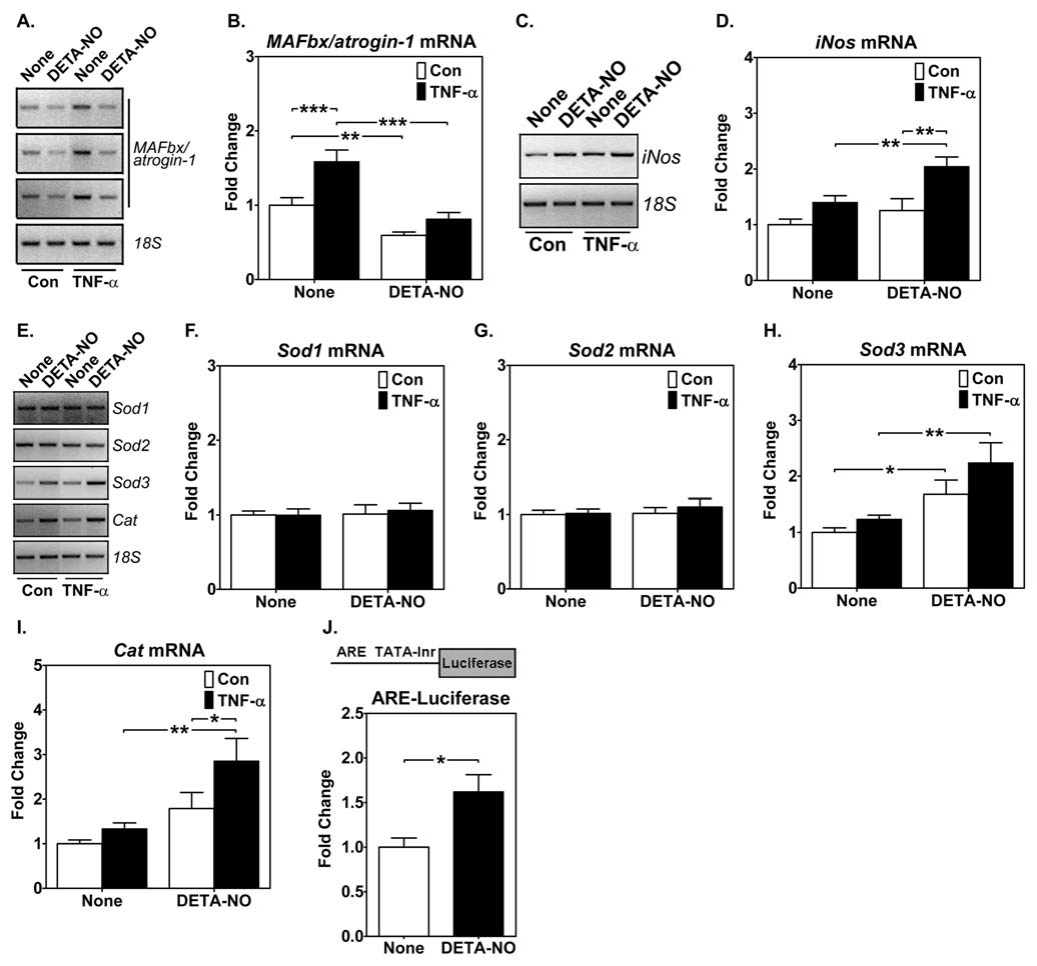
NO enhances *iNos* and antioxidant gene expression, and blocks TNF-α-induced *MAFbx/atrogin-1* mRNA expression in cultured myoblasts. Total RNA from cultured C2C12 myoblasts following treatment with NO donor, DETA-NO (0.2 mM), for 24 hours in the presence or absence of TNF-α (10 ng/ml) were assayed for *MAFbx/atrogin-1*, *iNos* and antioxidant gene expression by semi-quantitative RT-PCR and total protein lysates from transfected C2C12 myoblasts were harvested and assayed for luciferase activities. A) Gel images show levels of *MAFbx/atrogin-1* mRNA expression (in triplicates) with *18S* ribosomal RNA as control; B) Quantification and comparison of *MAFbx/atrogin-1* mRNA (n = 11; ** and *** *P*<0.01 and 0.001, respectively); C) Gel images show levels of *iNos* mRNA expression with *18S* ribosomal RNA as control; D) Quantification and comparison of *iNos* mRNA (n = 6; ** P<0.01); E) Gel images show levels of *Sod1*, *Sod2*, *Sod3 and Cat* mRNA expression with *18S* ribosomal RNA as control; F), G), H) and I) Quantification and comparison of *Sod1*, *Sod2*, *Sod3 and Cat* mRNA, respectively (n = 6; * and ** P<0.05 and 0.01, respectively); and J) pARE-Luc reporter gene activity in C2C12 myoblasts treated with or without DETA-NO for 24 hours (n = 6; * P<0.05).

Our findings that there are greater increases in NO production and antioxidant gene expression in oxidative than glycolytic muscles in response to cachectic stimuli suggest a functional link between NO production and the antioxidant defense system. To test this hypothesis, we cultured mouse C2C12 myoblasts with TNF-α in the presence and absence of NO donor, diethylenetriamine NO (DETA-NO). DETA-NO resulted in a moderate, but significant, reduction in the basal level expression *MAFbx/atrogin-1* mRNA and completely blocked the induction of *MAFbx/atrogin-1* induced by TNF-α (Fig. 4A and 4B). DETA-NO enhanced TNF-α-induced *iNos* expression significantly (Fig. 4C and 4D). These findings indicate that NO is sufficient to reduce cytokine-induced activation of the proteosome-dependent catabolic pathway, which appears to be mediated by iNOS. To determine if NO induces antioxidant gene expression, we performed semi-quantitative RT-PCR analysis for *Sod1*, *Sod2*, *Sod3* and *Cat*. DETA-NO increased the basal level and TNF-α-induced *Sod3* and *Cat* expression, whereas Sod1 and Sod2 were not responsive (Fig. 4E–4I), suggesting that *Sod3* and *Cat* play an important role in mitigating oxidative stress induced by cytokines in myocytes. To further confirm that these findings are due to NO-dependent transcriptional activation of the antioxidant genes, we transfected C2C12 myoblasts with the antioxidant response element (ARE) TATA-Inr luciferase reporter gene, pARE-Luc [29], and treated the myoblasts with or without DETA-NO for 24 hours [30]. The reporter gene expression was significantly stimulated by NO donor, suggesting that NO stimulates antioxidant genes through the *cis*-acting ARE DNA sequences.

### S-nitrosoglutathione (GSNO) blocks endotoxin-induced muscle-specific E3 ligase expression and enhances iNOS and antioxidant gene expression in skeletal muscle *in vivo*

**Figure 5:**
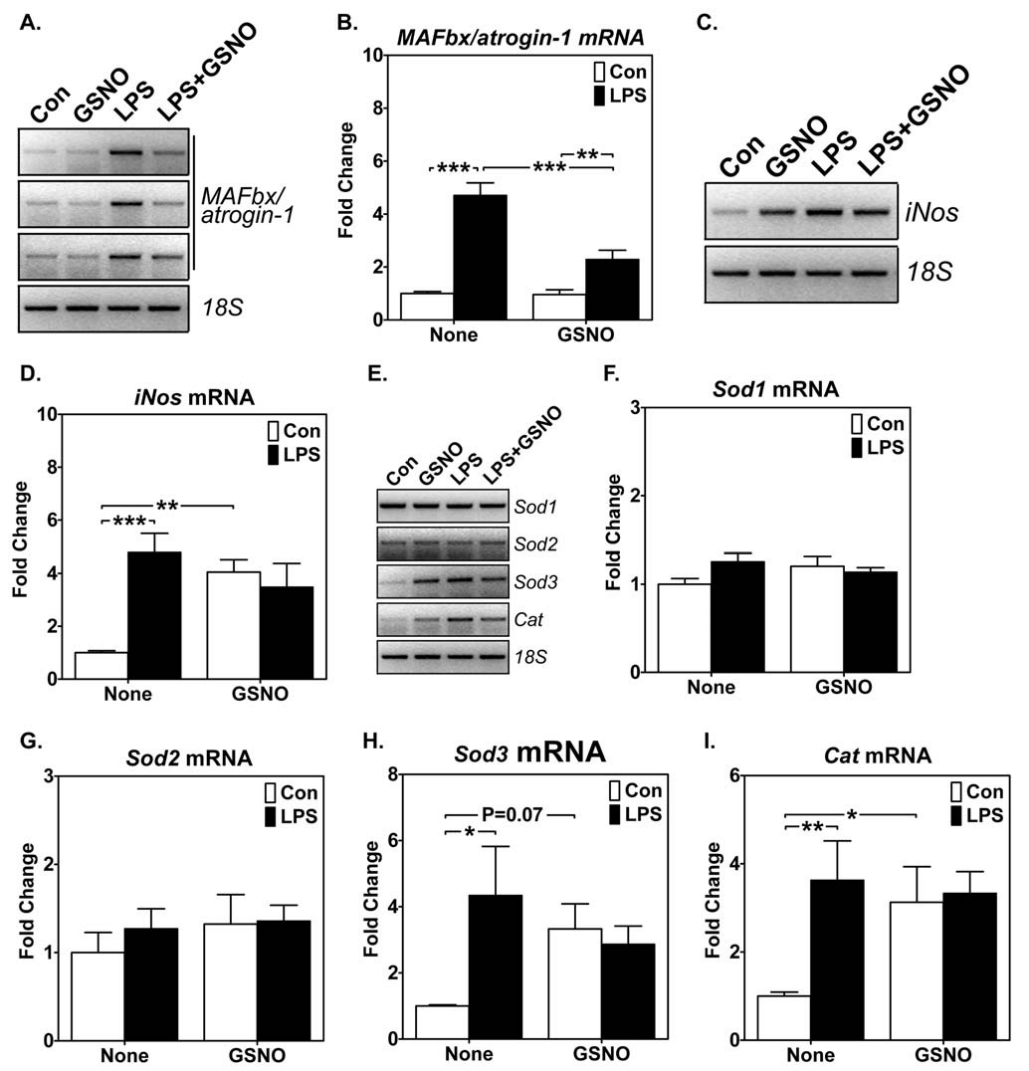
GSNO enhances *iNos* and antioxidant gene expression, and blocks LPS-induced *MAFbx/atrogin-1* mRNA expression in mouse plantaris muscles. Total RNA following NO donor GSNO injections (1 mg/kg, i.p. twice) in mice with LPS (1 mg/kg) or saline injection (Con) were assayed for *MAFbx/atrogin-1*, *iNos* and antioxidant enzyme mRNA expression by semi-quantitative RT-PCR. A) Gel images show levels of *MAFbx/atrogin-1* mRNA expression (in triplicates) with *18S* ribosomal RNA as control; B) Quantification and comparison of *MAFbx/atrogin-1* mRNA (n = 11; ** and ***P<0.01 and 0.001, respectively); C) Gel images show levels of *iNos* mRNA expression with *18S* ribosomal RNA as control; D) Quantification and comparison of *iNos* mRNA (n = 6; ** and *** P<0.01 and 0.001, respectively); E) Gel images show levels of *Sod1*, *Sod2*, *Sod3 and Cat* mRNA expression with *18S* ribosomal RNA as control; and F), G), H) and I) Quantification and comparison of *Sod1*, *Sod2*, *Sod3 and Cat* mRNA, respectively (n = 6; * and ** P<0.05, and 0.01, respectively).

The findings in cultured muscle cells motivated an *in vivo* study to confirm the functional role of NO in intact skeletal muscle. We injected the mice with the endogenous NO donor, GSNO (1 mg/kg, i.p.; 6 hours before and immediately before LPS injection) and performed semi-quantitative RT-PCR analysis in plantaris muscles (a muscle with a mixture of both oxidative type IIa and glycolytic type IId/x and IIb fibers). GSNO effectively blocked *MAFbx/atrogin-1* mRNA expression induced by LPS (Fig. 5A and 5B) and promoted *iNos* mRNA expression both with and without LPS injection (Fig. 5C and 5D). Therefore, NO protects myofibers from endotoxin-induced oxidative stress and prevent activation of the proteosome-dependent catabolic pathway, possibly through induced expression of iNOS. RT-PCR analysis for *Sod1*, *Sod2*, *Sod3* and *Cat* in plantaris muscles showed that GSNO resulted in increases in *Sod3* and *Cat*, but not *Sod1* and *Sod2*, expression with and without LPS injection (Fig. 5E–5I). These findings were entirely consistent with the findings in cultured myoblasts, suggesting the importance of SOD3 and CAT in NO-mediated protection against cachetic stimuli.

## Discussion

The mechanisms underlying the protection of oxidative myofibers against cachexia have been unclear. The major findings of this study are: 1) Protein oxidation and degradative protein (muscle-specific E3 ubiquitin ligase) expression were lower in oxidative than in glycolytic muscles in response to cachectic stimuli; 2) iNOS expression, NO production and antioxidant gene expression were higher in oxidative muscles than in glycolytic muscles; and 3) NO donors enhanced iNOS and antioxidant gene expression and attenuated atrophic muscle responses both *in vitro* and *in vivo*. These findings together strongly suggest that NO-dependent up-regulation of the antioxidant genes, at least partly mediated by iNOS, protects oxidative myofibers against cachectic stimuli. The studies not only provide potential mechanistic insights into the functional protection in oxidative muscle fibers, but also suggest the therapeutic value of enhanced NO signaling to the treatment of muscle wasting.

The fiber type-specific muscle wasting could be due to an intrinsic difference(s) in the oxidant/antioxidant system. We have recently shown that oxidative myofibers are protected from cachectic stimuli, such as LPS and TNF-α [12]. The finding in this study that endotoxin resulted in significantly less protein oxidation in oxidative muscle than in glycolytic muscle (Fig. 1A and 1B) is consistent with the notion that a more robust basal and/or inducible antioxidant system in oxidative myofibers prevents ROS from accumulating to a high level causing cellular damage. In fact, it has been shown that oxidative muscles have more robust expression and activity of antioxidant enzymes than glycolytic muscles [31], [32], and there is less mitochondrial superoxide production in oxidative myofibers than glycolytic myofibers [33]. Our findings of enhanced antioxidant gene expression in oxidative muscles (Fig. 3A–3E) suggest that an inducible antioxidant system provides additional protection against muscle catabolism. Elucidation of the mechanism responsible for such an inducible defense system will likely lead to the discovery of new drug target(s) for cachexia.

We also found that oxidative muscles produce more NO than glycolytic muscle and exhibit attenuated atrophic responses following endotoxin challenge (Fig. 2A), raising the possibility of a cause-effect relationship between NO and muscle protection. Paradoxically, it has been shown that NO and RNS inhibit mitochondrial respiration and muscle contractile function [21], [25]–[27], which could be prevented by ROS-RNS scavengers [23] or NOS inhibitors [25]. The interpretation was that iNOS mediated NO production was detrimental to muscle wasting. These previous findings were obtained in *ex vivo* experiments focusing on the acute effect of NO on muscle metabolic and contractile functions. Therefore, the mechanism(s) by which NO may protect against long-term muscle injury is currently not known. Our fiber type-specific analysis provides exciting new insight, suggesting induced NO production may play a protection against cachectic stimuli, rather than mediate muscle wasting.

NO production in skeletal muscle could involve nNOS (NOS1), iNOS (NOS2) and/or eNOS (NOS3) as they have been detected in skeletal muscles. A recent study showed that LPS injection resulted in increased NO production and changes in redox state in skeletal muscle, which were absent in *iNos* ^−/−^ mice [23], demonstrating the relevance of iNOS in muscle wasting. Here, we observed that induced iNOS expression was associated with increased NO production in oxidative muscle that was protected from endotoxin challenge (Fig. 2A, 2B, 2C and 2F). In contrast, endotoxin resulted in decreased *nNos* expression in both oxidative and glycolytic muscles (Fig. 2B and 2D). nNOS protein is specifically expressed in glycolytic myofibers as part of dystrophin glycoprotein complex [34] that maintains skeletal muscle contractile function [35], [36]; reduced *nNos* expression here is consistent with a general sarcolemmal injury [36]. On the other hand, induced *eNos* expression in glycolytic myofibers (Fig. 2B and 2E) does not reconcile with the protection in oxidative myofibers. Taken together, cachexia-induced NO production in skeletal muscle appears to be mediated through enhanced iNOS expression. The apparent feed-forward regulation of NO production may prove to be critical in amplifying the signals necessary for the protection associated with oxidative phenotype.

Paradoxically, melatonin, a powerful scavenger of both ROS and RNS, abolished the increases in NO production and GSSG/GSH ratio in isolated mitochondria from skeletal muscles [23] and heart [37] in mice challenged with endotoxin; these increases were absent in *iNos^−/−^* mice. Our confined staining of oxidative myofibers of iNOS to the periphery is most consistent with mitochondrial localization of iNOS protein, which is in agreement with a recent finding [23]. The previous findings that cachetic responses were absent in *iNos^−/−^* mice were interpreted as evidence that iNOS was responsible for mitochondrial dysfunction and oxidative stress in muscles wasting. However, precaution should be taken regarding these findings with overall inhibition of iNOS since whole body genetic disruption of the *iNos* gene and application of iNOS inhibitor leads to loss of iNOS function not only in skeletal muscles, but also in macrophages, which is critical for the induction of sepsis [38]. On the other hand, a powerful protective function of iNOS in skeletal muscle are suggested in previous studies using a rat model of myasthenia gravis [11] caused by autoantibodies binding to and inhibiting the nicotinic acetylcholine receptors at the neuromuscular junctions. iNOS inhibitor converted disease-resistant soleus muscle to a disease-susceptible phenotype [11]. In this study, both *in vitro* and *in vivo* findings provide strong evidence support the protective function of NO in skeletal muscle in cachexia. Nevertheless, a formal confirmation of iNOS function in skeletal muscle awaits the generation of an animal model of skeletal muscle-specific knockout of the *iNos* gene.

We observed significant enhanced antioxidant genes in oxidative soleus muscles, but not in glycolytic white vastus lateralis muscles (Fig. 3A–3E), a first demonstration of fiber type-specific induction of antioxidant genes in a whole animal model of muscle wasting. We then addressed the question whether enhanced NO production/signaling renders protection against cachetic stimuli induced by endotoxin/cytokine. Both *in vitro* and *in vivo* findings suggest that NO reduces atrophic responses and enhances antioxidant gene expression in skeletal muscles (Fig. 4 and 5). To our knowledge, this is the first demonstration of simultaneous, fiber type-specific induction of *Sod3* and *Cat*. Based on these findings, we now conclude that oxidative myofibers possess an intact NO-dependent signaling and transcription system protecting the myofibers from oxidative stress by enhancing the antioxidant system. The finding of enhanced reporter gene activity in cultured muscle cells transfected with the ARE reporter gene (Fig. 4J) further supports this conclusion and provides more direct evidence of transcriptional control. Further research should focus on the link between the NO signal and the transcriptional factors in skeletal muscle that is responsible for the inducible defense system.

It is worth noticing that NO donor was sufficient both in vitro and in vivo to induce iNOS expression (Fig. 4C and 4D and Fig. 5C and 5D), suggesting that both culture muscle cells and skeletal muscles have intact machineries to induce iNOS expression; the difference may be that oxidative muscle, but not glycolytic muscle, has the ability to trigger the initial increase in NO production. Future experiments should focus on elucidating the mechanism underlying this important regulatory event.

Accumulating evidence supports the view that NO and RNS play protective functions in various tissues/organs and disease models [39]–[41]. Perhaps, the most relevant findings are in the heart [42], [43] through an iNOS-dependent mechanism [44] as cardiac muscle has metabolic and contractile functions that more closely resemble oxidative skeletal muscles. More recently, a calcineurin/NFAT (nuclear factor of activated T-cells) signaling cascade has been implicated in cardiomyocyte protection through the transcriptional control of the *iNos* gene [45]. Ours is the first report that NO and RNS play a protective role against cachetic stimuli in skeletal muscle. We believe that NO elicits signaling events leading to the transcriptional activation of the antioxidant genes though the exact signaling and molecular mechanisms responsible for the up-regulation of the antioxidant genes remain to be elucidated.

**Figure 6:**
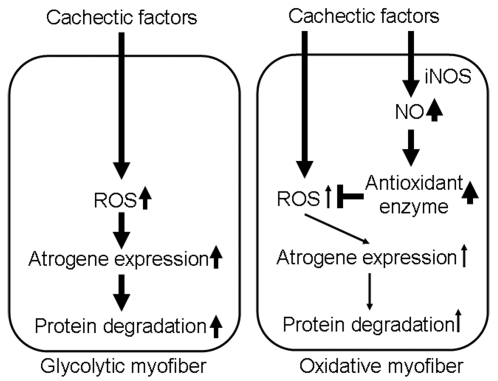
Schematic presentation of a novel framework for oxidative phenotype-associated protection against muscle wasting. Cachectic factors increase ROS in glycolytic myofibers; oxidative stress in turn results in up-regulation of atrophic genes (atrogenes), enhances protein degradation and muscle atrophy. ROS are reduced in oxidative myofibers due to expression of iNOS and NO-dependent up-regulation of the antioxidant genes, leading to less atrogene expression and protein degradation. Thick and thin arrows represent great and little increase/stimulation, respectively.

In summary, findings from this study are consistent with a hypothesis as following. Cachexia leads to increased oxidative stress and consequently activation of protein degradation pathways in glycolytic myofibers. Enhanced iNOS expression and NO production lead to enhanced antioxidant gene expression in oxidative myofibers, providing protection against oxidative stress (Fig. 6). These findings suggest a therapeutic value of enhancing NO signaling in skeletal muscle for cachexia. Future investigations should ascertain the functional role of enhanced NO signaling in protection against various types of muscle atrophy and elucidate the molecular mechanism(s) responsible for the fiber type-specific expression of *iNos* and antioxidant genes. It should be noted that skeletal muscle wasting during cachexia could be affected by protein synthesis, protein degradation, autophagy and other processes. The finding of linking NO-dependent inducible antioxidant system to reduced atrogene expression may represent one of the mechanisms of endogenous defense systems in skeletal muscle under the condition of cachectic challenges.

## Materials and Methods

### Experimental animals

Male C57BL/6 mice (8–9 weeks old) from the Jackson Laboratory (Bar Harbor, ME) were maintained in light- (12∶12 h light-dark cycle) and temperature-controlled quarter (21°C) provided with water and chow (Purina, Richmond, IN) *ad libitum*. They were intraperitoneally injected (i.p. 1 mg/kg) with *E. coli* LPS (Sigma, St. Louis, MO) or normal saline 12 hours before being sacrificed by isoflurane-induced anesthesia and cervical dislocation. LPS injection did not result in morbidity or mortality. Soleus and white vastus lateralis muscles were harvested. To determine the role of NO donor *in vivo*, GSNO (Sigma, St. Louis, MO) were injected (i.p. 1 mg/kg) twice (6 hours prior to and at the time of LPS or normal saline injection). Plantaris muscles were harvested 12 hours later. All animal protocols were approved by the Duke University Institutional Animal Care and Use Committee.

### Protein carbonylation assay

To determine the level of oxidative stress in skeletal muscle, carbonyl levels of proteins were determined in whole muscle lysates by using the OxyBlot™ Protein Oxidation Detection Kit (Serologicals Corporation, Norcross, GA). Briefly, harvested skeletal muscles were placed in 5-ml Falcon tubes each containing 1 ml of ice-cold Protein Lysis Buffer (Cell Signaling Technology, Danvers, MA) supplemented with 50 mM dithiothreitol (DDT) and 1 mM phenylmethylsulphonyl fluoride (PMSF) and homogenized with an Ultra Turrax T25 Polytron™ homogenizer. The homogenates were centrifuged at 16,000 × g for 5 min at 4°C, and the supernatants were then transferred to 10K NMMWL-0.5 ml Ultrafree Filter Unit (Millipore, Bedford, MA) and centrifuged at 16,000 × g at 4°C (about 30 min) to concentrate to a volume of about 60 µl. Protein concentration was then determined by using the RC DC protein assay (BioRad, Hercules, CA). Protein carbonylation was then determined in the whole muscle lysate (20 µg protein) by an immunblot-based assay according to the instructions from the manufacturer. The intensities of all the bands in each of the lanes were quantified by using Scion Image (Scion Corporation, Frederick, Maryland) and normalized by α-tubulin.

### Nitrite assay

Nitrite was assessed using the Griess Reagent System (Promega, Madison, WI). In brief, harvested skeletal muscles were homogenized immediately in ice-cold Protein Lysis Buffer (Cell Signaling Technology, Danvers, MA) containing 1 mM PMSF in a Wheaton glass-on-glass homogenizer on ice, and the homogenate was centrifuged at 16,000 × g for 5 min at 4°C. The protein concentration of the supernatants was determined as described above. The nitrite assay was started by adding the sample (360 µg proteins in 50 µl) to 50 µl sulfanilamide solutions in 96-well plates in duplicate. After incubation at room temperature for 10 min, 50 µl of the N-1-napthylethylenediamine dihydrochloride (NED) solution was added, and the reaction was continued for another 10 min. Absorbance at 550 nm was then measured, and nitrite concentration was calculated after normalization by total protein.

### Cell culture

C2C12 mouse myoblasts were grown and maintained as sub-confluent monolayer in high-glucose Dulbecco modified Eagle medium (DMEM; Gibco BRL, Grand Island, NY) supplemented with 20% fetal bovine serum (FBS, Gibco BRL, Grand Island, NY). To examine the effect of NO donor and iNOS inhibitor, C2C12 cells seeded in 35 mm-wells at 1 × 10^5^ and allowed to adhere for at least 12 hour prior to stimulation by TNF-α (R&D Systems, Minneapolis, MN) at 10 ng/ml with/without DETA-NO (Sigma, St. Louis, MO). The cells were harvested 20 hours later, and total RNA samples were isolated for RT-PCR analysis by using Trizol (Invitrogen, Carlsbad, CA). For transfection of pARE-Luc, C2C12 myoblasts were transfected with 0.5 µg with Lipofectamine 2000 according the manufacturer's instructions. After overnight transfection, the transfected cells were treated with/without DETA-NO (10 ng/ml) for 24 hours. Luciferase activity was assayed and is expressed as relative activity (fold change) in comparison to the value of transfected cells without DETA-NO. The transfection was done twice with triplicate samples for each condition.

### Semi-quantitative RT-PCR

This analysis was performed as described [12] to measure *MAFbx/atrogin-1*, *MuRF1*, *iNos*, superoxide dismutases (*Sod-1*, *Sod-2*, *Sod-3*) and catalase *(Cat)* mRNA expression in skeletal muscles and in cultured C2C12 myoblasts. PCR primer pairs were designed using Primer3 search engine at www-genome.wi.mit.edu. The oligonuleotide primer pairs used in this study corresponded to the following nucleotides: *MAFbx/atrogin-1*: 1917–1936 and 2415–2396 (NM_026346); *MuRF1*: 50–69 and 452–433 (AF294790); *iNos*: 1815–1834 and 2301–2282 (MMU43428); *eNOS:* 1515–1534 and 2002–1983 (NM_008713); *nNOS:* 1261–1272 and 1735–1716 (NM_008712); *Sod1:* 168–187 and 583–564 (NM_011434); *Sod2*: 1218–1237 and 1647–1628 (BC066063); *Sod3*: 1547–1566 and 2019–2000 (NM_011435); and *Cat*: 237–256 and 645–626 (MMU43428). Results were normalized by *18S* rRNA and presented as fold change to soleus muscle in mice injected with normal saline.

### Statistics

Data are presented as mean±standard error. For comparisons involving two factors, two-way ANOVA was performed followed by the Newman-Kuels test. For comparisons between two groups, the Student t-test was performed. *P*<0.05 was accepted as statistically significant for all the experiments in this study.
